# Role of priority effects in invasive plant species management: Early arrival of native seeds guarantees the containment of invasion by Giant ragweed

**DOI:** 10.1002/ece3.9940

**Published:** 2023-03-26

**Authors:** Chaeho Byun

**Affiliations:** ^1^ Department of Biological Sciences Andong National University Andong 36729 Korea

**Keywords:** additive competition design, *Ambrosia trifida*, biological resistance, competition experiment, invasion management, invasive plants, niche preemption, sowing time

## Abstract

Empirical evidence shows that early arrival of native species, which induces the priority effects, can contribute to invasive plant species containment. However, more systematic studies are required to test the applied relevance of the priority effect. This study therefore aimed at testing the priority effects generated by different sowing times of seeds of nine native species on one target invasive plant species, that is, Giant ragweed (*Ambrosia trifida*). This study hypothesized that, when sown earlier, some native species will be able to substantially contain *A. trifida* through resource preemption. An additive competition design was used to test the competitive effects of native species on *A. trifida*. Depending on the sowing times of native and invasive plant species, three priority treatments were conducted: all species sown at the same time (T1); native species sown 3 weeks before *A. trifida* (T2); and native species sown 6 weeks before *A. trifida* (T3). Priority effects created by all nine native species significantly affected the invasibility of *A. trifida*. The average value of the relative competition index (RCI_avg_) of *A. trifida* was the highest when native seeds were sown 6 weeks early and decreased with decreasing early sowing time of native plants. The species identity effect was not significant on RCI_avg_ if natives were sown at the same time or 3 weeks earlier than *A. trifida* invasion, but it was significant (*p* = .0123) if they were sown 6 weeks earlier than *A. trifida*. *Synthesis and applications.* The findings of this study clearly show that native species, when sown early, provide strong competition and resist invasion through prior utilization of resources. The consideration of this knowledge might improve *A. trifida* invasion management practices.

## Graphical abstract

Depending on the sowing times of native and invasive plant species, three priority treatments were conducted: all species sown at the same time (T1); native species sown 3 weeks before *Ambrosia trifida* (T2); and native species sown 6 weeks before *A. trifida* (T3). Priority treatments significantly affected the average response of *A. trifida* to all nine native species (*F*_2,76_ = 77.97, *p* < .0001). The average value of the relative competition index (RCIavg) of *A. trifida* was the highest in T3 (least squared mean [LSD] = 0.695), followed by T2 (LSD = 0.308), and T1 (LSD = 0.041).

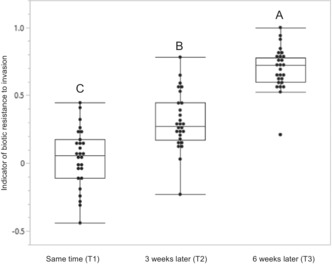

## 1 INTRODUCTION

Biotic resistance to invasion refers to the ability of native communities to limit invasion success through a biological process, such as competition (Levine et al., 2004; Yannelli, 2021). One of the reasons for the vulnerability of native plant communities to invasion is the lack of biological resistance (Ibáñez et al., 2021). Resident plants employ biotic resistance to control invasive species spread. Biotic resistance of native plants can also be used to design competitive plant communities to prevent the establishment and dominance of an alien invasive plant and/or to outcompete an already‐established alien plant species (Guo et al., 2019; Kettenring & Adams, 2011; Tarsa et al., 2022; Weidlich et al., 2020). Biotic resistance through competition to invasive species is determined by many factors, including limiting similarity (Hess et al., 2020; Price & Pärtel, 2013), functional traits or functional groups (Drenovsky et al., 2012; Hooper & Dukes, 2010), diversity effects (Byun, de Blois, & Brisson, 2020; Henriksson et al., 2016), seed sowing density (Byun, Oh, et al., 2020; Tarsa et al., 2022), and priority effects (Weidlich et al., 2021). Among these, the role of priority effects (or niche preemption), use of resources by early‐arriving species and thereby affecting the performance of late arrivals (Fukami, 2015), to control invasion is gaining attention of scientific community to create new knowledge and its practical application. Priority effects are determined by the order or timing of species arrival; species that arrive early at a site affect, either positively or negatively, the establishment, growth, or reproduction of species that arrive at subsequent time points (Weidlich et al., 2021). Recent empirical evidence demonstrates that invasive species rely on the early and effective utilization of resources to germinate, establish, grow, and maintain dominance (Byun, 2022; Hess et al., 2019; Weidlich & de Dechoum, 2021; Yannelli et al., 2020). Such a situation can also be developed for native plant species to enhance their resistance against invasion, although additional systematic studies are needed to investigate the potential of early‐arriving native species in limiting the spread of invasive species (Hess et al., 2019; Yannelli et al., 2020).

Functional traits of a species refer to its morpho‐physio‐phenological characteristics (Cornelissen, 2003; Violle et al., 2007), and functional trait similarity between native and invasive species is expected to lead to niche overlap and therefore competition (Funk et al., 2008). Functional group, based on functional trait similarity, can be an important determinant of biotic resistance to invasion (Byun et al., 2013; Gooden & French, 2015; Sheley & James, 2017). It is, therefore, essential to classify species into several functional groups based on relevant traits to relate functional group identity with biotic resistance to invasion.

Giant ragweed (*Ambrosia trifida* L., hereafter *A. trifida*) is a notorious alien species worldwide (Brandes & Nitzsche, 2006; Byun, Choi, & Kang, 2020; Byun & Lee, 2018; Page & Nurse, 2015) and is listed as a harmful invasive plant species in South Korea (Kil et al., 2004). Ecologically, *A. trifida* is considered as one of the most destructive weeds (Kong et al., 2007; Quinn et al., 2021) that results in several harmful consequences, such as diversity loss, habitat degradation, and health problems (pollen allergy; Harrison et al., 2001; Kim et al., 2018; Wang et al., 2022; Washitani, 2001; Yin et al., 2010). In natural habitats, *A. trifida* dominates the community in which it is present by suppressing the growth of all other plant species, thus accounting for most of the plant biomass (Abul‐Fatih & Bazzaz, 1979). *A. trifida* is increasingly becoming a major problem in agriculture because it outcompetes economically important plant species, such as corn and soybean, and reduces grain yield (Brandes & Nitzsche, 2006; Harrison et al., 2001; Quinn et al., 2021). However, in a recent study conducted in central Europe, the growth of *A. trifida* did not create any competition for other plant species (Savić et al., 2021).

*Ambrosia trifida* is very difficult to control owing to not only its rapid growth but also its ability to produce a large number of seeds that can easily spread to geographically distant areas via a range of dispersal methods and can germinate under stressful conditions (Byun, Choi, & Kang, 2020; Byun & Lee, 2018; Wang et al., 2022). Prolonged and continuous invasion by *A. trifida* can alter the local seed bank composition and substantially decrease the abundance of native seeds (Wang et al., 2022). The on‐site restoration of native communities intensively dominated by *A. trifida* seeds generally requires robust efforts. Lack of native seeds in the soil seed bank makes restoration even more difficult because *A. trifida* can easily reinvade disturbed sites (i.e., bare ground) after the application of eradication methods. Although various physical and chemical control methods have been employed, modest success has been achieved in a few studies (Ganie et al., 2016; Kaur et al., 2014; Quinn et al., 2021). In an experimental field study, manually cutting the *A. trifida* plants was determined as the most effective method of control, but sowing the seeds of native species in addition to manual cutting did not have an additional benefit to invasion control (Byun, Choi, & Kang, 2020). This was most likely because of the late arrival of native seeds in an already invaded site with a robust *A. trifida* legacy. In another study, biotic resistance was not effective in controlling *A. trifida* (Byun & Lee, 2018); however, these authors did not test different times of arrival of native species and invasion by *A. trifida*. The tested mechanisms are relevant to preventing the establishment of *A. trifida* not to controlling an existing population. Reducing invasion or preventing the establishment of invasive species must be coupled with increasing the priority effects of native plant communities.

Considering these research gaps, the current study tested the effectiveness of priority effects created by native species in preventing invasion by *A. trifida* and containing its spread and invasion. The priority effects of native species were generated by sowing *A. trifida* seeds at different times after the arrival of native species. This study hypothesized that native species can substantially suppress invasion by *A. trifida* because of the priority effects created by their early arrival. This study also hypothesized that certain plant functional groups, such as annuals, nonwoody perennials, and woody plants, would exhibit the strong resistance to *A. trifida* invasion. This implies that biotic resistance to invasion by *A. trifida* would be determined by the functional group identity of tested native species. This study addresses how the identity and priority effects of native species affect the success of invasive species.

This study investigates, for the first time, the potential role of priority effects of multiple native species in controlling *A. trifida*, an invasive exotic species. In addition, this study demonstrates how the identity and priority effects of native species can decrease invasion by *A. trifida*.

## 2 MATERIALS AND METHODS

### 2.1 Native species selection and functional group classification

Biotic resistance to invasion by *A. trifida* was assessed among nine species native to Republic of Korea. These native species included three annuals (*Lactuca indica* L., *Commelina communis* L., and *Persicaria hydropiper* [L.] Delarbre), three herbaceous perennials, (*Pennisetum alopecuroides* [L.] Spreng., *Dianthus chinensis* L., and *Plantago asiatica* L.), and three woody perennials (*Lespedeza bicolor* Turcz., *Lespedeza cuneata* [Dum.Cours.] G.Don, and *Sorbaria sorbifolia* [L.] A.Braun). These nine species were selected based on their habitat preference (shared with *A. trifida*), seed availability (local seed suppliers), germination rate (>5%), and nativeness to the study region. The species nomenclature used in this study was obtained from The Plant List (http://www.theplantlist.org), which showed the accepted name by Flora of Korea Editorial Committee (2007).

### 2.2 Experimental setup and seed preparation

A pot experiment was established in the greenhouse facility of the Department of Biological Sciences and Biotechnology at Andong National University. The experiment was designed to simulate a scenario where seeds of *A. trifida* and native plants arrive on bare fertile soil at different times after a biological and human disturbance.

Seeds of *A. trifida* were collected from five different populations in the natural field of riverside habitats of Gyeongancheon Stream (Yongin City, Gyeonggi Province, Republic of Korea), and seeds of all native plant species were purchased from local seed suppliers. Both native and invasive seeds were arranged during the winter of 2021. To determine seed viability, all seeds were cold stratified at 3°C, as previously described (Byun, Oh, et al., 2020). To test seed germination, 100 seeds of each species were placed in a Petri dish lined with filter paper (Whatman® No. 1), which was moistened daily with 6 mL of distilled water. The plates were incubated under fluorescent light for 5 weeks. Germination tests were conducted in three replicates for each species. All species showed >5% germination rates. Seeds (not seedlings) of each species were sown in pots (22 cm diameter, 30 cm height) filled with commercial fertile agricultural soil.

### 2.3 Experimental design

An additive design was used to test the competitive effect of native species on *A. trifida* (Connolly et al., 2001; Keddy et al., 1994; Snaydon, 1991). Three treatments were conducted to test the priority effects of native species: (1) sowing native and invasive species at the same time (T1); (2) sowing native species 3 weeks before *A. trifida* (T2); and (3) sowing native species 6 weeks before *A. trifida*. In the T1 treatment, the seeds of native species and *A. trifida* were sown on March 18, 2022; in T2, the seeds of native species were sown on March 18, 2022, and those of *A. trifida* were sown on April 8, 2022; and in T3, the seeds of native species were sown on March 18, and those of *A. trifida* were sown on April 29, 2022. Each priority treatment was conducted in 10 experimental pots; 9 pots were sown with the viable seeds of nine native species and *A. trifida* at a ratio of 2.5:1 (20 seeds of native species and 8 seeds of *A. trifida*), and one pot (control) was sown with *A. trifida* seeds only. Each treatment was further replicated three times. Thus, a total of 90 experimental pots (3 treatments × 10 pots per treatment × 3 replications) were established. Pots were arranged in a randomized complete block design. Soil water content was maintained in each pot using water drip irrigation.

### 2.4 Data collection and analysis

Performance traits of *A. trifida* plants including shoot number per plant (shoot density), plant height, and canopy cover were measured biweekly in treatment and control pots to calculate the primary response variable (see Equations (1) and (2) below). The shoot number per plant (shoot density), plant height, and canopy cover of native species were also measured. Subsequently, the traits of native species were compared and correlated with the main response variable (see Equation (2) below). Plant height for each species was estimated to the closest 0.5 using the tallest branch as the maximum height. Canopy cover (%) was estimated by visually assessing the relative area covered by the different species in each pot. In mid‐August, when all species reached maturity, the aerial parts of native and *A. trifida* plants were harvested, weighed, and then dried at 80°C for 48 h to determine their aboveground biomass.

Relative competition index (RCI), which measures the competitive effect of native species on invasive species, was calculated using the following equation (Weigelt & Jolliffe, 2003):

(1) $${\mathrm{RCI}}_{Y}=\frac{Y_{\text{control}}-Y_{\text{treatment}}}{Y_{\text{Control}}}$$

where RCI_Y_ is the RCI of *A. trifida* for a given variable *Y* (aboveground biomass, plant height, shoot density, or canopy cover); *Y*_control_ is the performance of *A. trifida* in the control pot; and *Y*_treatment_ is the performance of *A. trifida* in a treatment.

Because RCI_biomass_, RCI_height_, RCI_shoots_, and RCI_canopy cover_ were highly correlated with one another, the average RCI (RCI_avg_) was calculated as the primary response variable for all treatments using the following equation:

(2) $${\mathrm{RCI}}_{\mathrm{avg}}=\frac{\left({\mathrm{RCI}}_{\text{biomass}}+{\mathrm{RCI}}_{\text{height}}+{\mathrm{RCI}}_{\text{shoots}}+{\mathrm{RCI}}_{\text{cover}}\right)}{4}$$

RCI_avg_ = 0 indicates no competitive effect on *A. trifida*; RCI_avg_ = 1 indicates complete competitive exclusion of *A. trifida* (no invasion); and RCI_avg_ < 0 indicates facilitation of the establishment and growth of *A. trifida*.

### 2.5 Statistical data analysis

The effects of priority on RCI_avg_ were tested by performing the analysis of variance (ANOVA). A general linear mixed model (REML; *F*‐test) was used to account for the random block effect (Bolker et al., 2009). The normality and homoscedasticity of residuals were evaluated, and response variables were log‐transformed when necessary. When a significant effect was detected, Tukey's honestly significant difference (HSD) multiple comparison test was used to compare the means. Statistical significance was assessed using the *F* statistic and *p*‐value (<.05). Correlation between the performance traits and relative competition index of native species was tested as described previously (Byun et al., 2022; Byun, Oh, et al., 2020). All ANOVA tests and correlation analyses were conducted using the JMP software (SAS Institute Inc.).

## 3 RESULTS

**FIGURE 1:**
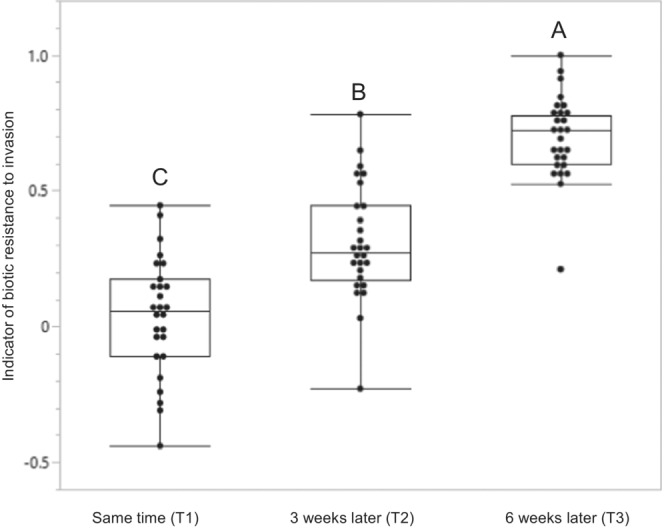
Effects of different sowing times o*f Ambrisoa trifida* (priority effect of native species) the averaged relative competition index (RCIavg), an indicator of biotic resistance of native species to invasion by *A. trifida*. The RCI_avg_ estimates the competitive effect of native species on *A. trifida* (see Section 2). Different letters indicate significant differences (*p* < .05; ANOVA).

This study hypothesized that, owing to their early arrival (priority effects), native species will effectively resist invasion by exotic species, that is, *A. trifida*. Consistent with this hypothesis, the effects of all three treatments (T1–T3) on the synthetic response variable (RCI_avg_, an indicator of biotic resistance to invasion) were significant (*F*_2,76_ = 77.97, *p* < .0001; Figure 1). RCI_avg_ was highest in the T3 treatment (*A. trifida* seeds sown 6 weeks after native species; least squared mean [LSD] = 0.695), followed by T2 (*A. trifida* seeds three weeks after native species; LSD = 0.308), and T1 (*A. trifida* and native species sown at the same time; LSD = 0.041; Figure 1).

**FIGURE 2:**
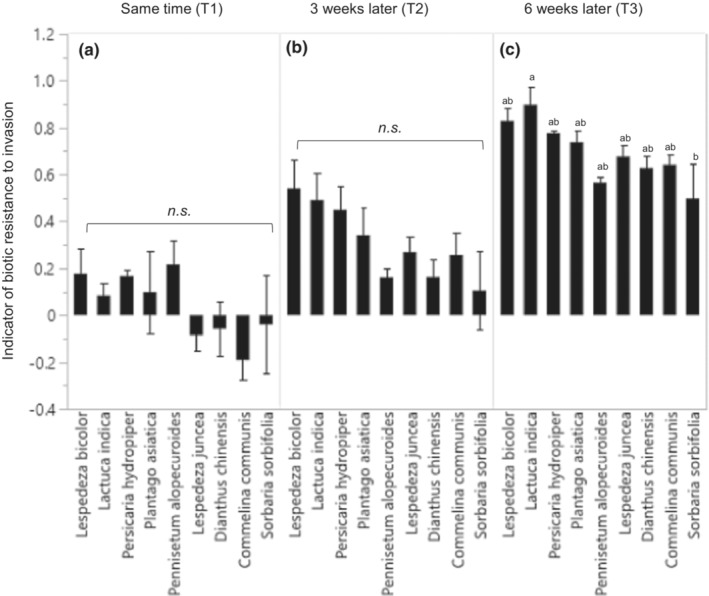
Effect of species identity on RCI_avg_, an indicator of biotic resistance to invasion by *Ambrisoa trifida*, in each treatment. Data represent mean ± standard error (SE) of three biological replicates. Different lowercase letters indicate significant differences (*p* < .05; ANOVA). (a) Priority effect treatments of same time (T1), (b) 3 weeks later (T2), (c) 6 weeks later (T3).

The effect of species identity on RCI_avg_ was significant in T3 (*F*_8,16_ = 3.70, *p* = .0123) but not in T1 (*F*_8,16_ = 1.64, *p* = .1879) and T2 (*F*_8,16_ = 2.21, *p* = .0831; Figure 2). Well‐established native species (sown 6 weeks early) were more effective in resisting invasion than native plants established later (sown 3 weeks early or at the same time as *A. trifida*). Among the nine species, the RCI_avg_ of only *L. indica* (mean = 0.898) was significantly different from that of *S. sorbifolia* (mean = 0.497). In addition, the effects of three functional groups (annuals, nonwoody perennials, and woody) on RCI_avg_ were not significant in T1 (*F*_2,22_ = 0.2994, *p* = .7442), T2 (*F*_2,22_ = 1.5907, *p* = .2263), and T3 (*F*_2,22_ = 1.5907, *p* = .2263).

**FIGURE 3:**
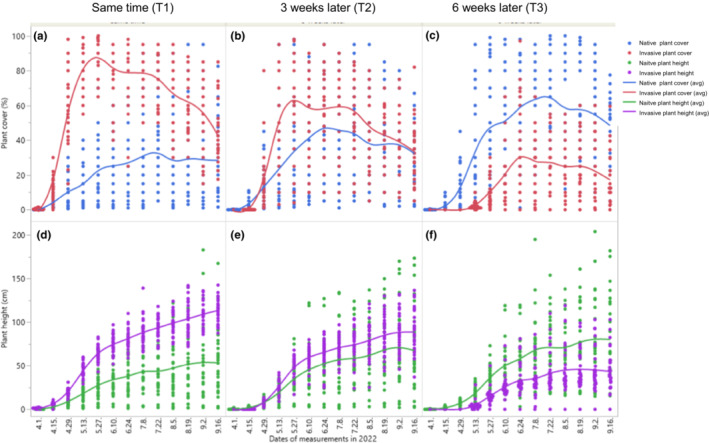
Biweekly changes in plant cover of native species (in blue color) and invasive species (red) and plant height of native species (green) and invasive species (purple) during the entire experimental period. (a) Priority effect treatments of same time (T1) for plant cover, (b) 3 weeks later (T2) for plant cover, (c) 6 weeks later (T3) for plant cover, (d) same time (T1) for plant height, (e) 3 weeks later (T2) for plant height, and (f) 6 weeks later (T3) for plant height.

Biweekly changes in the plant cover of native species and *A. trifida* showed distinctive patterns among treatments (Figure 3). The plant cover and plant height of native species were higher than those of *A. trifida* during the entire experimental period only in the T3 treatment. Photographs of pots containing the different species in each treatment were taken biweekly and are shown in Appendix S1.

**FIGURE 4:**
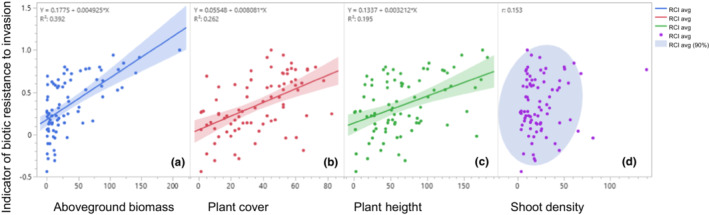
Relationship of the aboveground biomass (a), canopy cover (b), plant height (c), and density (d) of native species with RCI_avg_, an indicator of biotic resistance to invasion by *Ambrisoa trifida*. *r* represents the Pearson correlation coefficient.

The aboveground biomass, plant cover, and height of native species were significantly and positively correlated with RCI_avg_ (correlation coefficient [*r*] = .626, *p* < .0001 for biomass; *r* = .0.512, *p* < .0001 for plant cover; *r* = .442, *p* < .0001 for plant height), but shoot density was not significantly correlated with RCI_avg_ (*r* = .153, *p* = .1738; Figure 4).

## 4 DISCUSSION

The results of this study clearly demonstrate the benefits of priority effects (sowing native seeds earlier than invasive species) in controlling invasion by *A. trifida* (Figure 1). Native species arriving 6 weeks earlier than *A. trifida* were more effective in controlling invasion than those arriving 3 weeks earlier or at the same time as *A. trifida*. These results are consistent with observations made in previous studies on the priority effects in different plant systems, such as *Festuca pratensis*, *Plantago lanceolata*, *Knautia arvensis*, *Trifolium pratense*, *Rhamnus cathartica*, *Phragmites australis*, and California grasslands (Firn et al., 2010; Hess et al., 2019; Mwangi et al., 2007; Schuster et al., 2020; Tarsa et al., 2022; Weidlich & de Dechoum, 2021; Yannelli et al., 2020; Young et al., 2017). A study on priority effects, in which a native species was planted 4 weeks prior to the arrival of two invasive non‐native grasses (*Urochloa humidicola* or *U. decumbens*), reported poor development of invasive species (Evangelista et al., 2017). Similarly, the suppression of an invasive shrub *Cytisus scoparius* with higher productivity of native species *Festuca rubra* subsp. *ommutate* which arrived 21 and 29 days early was reported by Lang et al. (2017). The most likely mechanism responsible for this phenomenon is preemption (Fukami, 2015), that is, the early and effective utilization of resources by the early‐arriving species. This strategy, however, is generally weaker when employed by native species than when employed by invasive species (Dickson et al., 2012; Hess et al., 2019), affected germination and growth of *A. trifida* arrived late. Torres et al. (2022) also reported that late‐arriving non‐native species were less effective in dominating the community, because of limited availability of niche space and resources. However, some studies show that the priority effects of natives weaken over time (subsequent growing seasons), resulting in the increased dominance of competitive invasive species (Young et al., 2017).

Contrary to the results of this study and my hypothesis that early‐arriving natives belonging to different functional groups (grass, herbs, and shrubs) are more resistant to plant invasion, Mason et al. (2013) reported that the order of arrival does not affect resource use and invasion by a dominant exotic shrub *Chrysanthemoides monilifera* ssp. *Rotundata*, in coastal communities. This is likely due to the high immigration rate of invasive species and their adaptability to anthropogenically created systems (Brandt et al., 2017). In this study, few comparatively late‐arriving seeds of invasive species did not create competition for native species, most likely because of limited resource and space availability.

In other studies, researchers investigated whether the success of early‐arriving invasives species is attributable to their greater competitiveness for resource use or their earlier establishment compared with natives (Delory et al., 2019; Dickson et al., 2012; Goodale & Wilsey, 2018). Dickson and colleagues grew three pairs of invasive and native species (six species total) belonging to different functional groups at 3‐week intervals and found that the priority effects of invasive species are much stronger than that of natives. Similar trends were observed where pairs of native and invasive grass species were seeded 28 days early than arrival of mix of 39 native species. Results show that exotic priority effects can affect establishment and diversity of native communities more strongly than native priority effects (Goodale & Wilsey, 2018). Similarly, Delory et al. (2019) showed that the time of arrival is more beneficial for an exotic species than for a native grass community. However, Stuble and Souza (2016) reported that both native and invasive species can gain from arriving early, but invasive species were less affected by arriving late. The current study did not include a treatment where invasive species arrived earlier than natives; however, other studies have shown that natives pay more for late arrival than invasives (Torres et al., 2022). On the contrary, invasive species arriving late may germinate early to take hold on the community in subsequent years. Therefore, more long‐term studies are needed to understand the species‐specific priority effects under different environmental conditions (Kettenring & Tarsa, 2020).

This study provides strong evidence to prove the importance of priority effects in creating biotic resistance against invasion by *A. trifida*. Priority effects are also supported by the relatively high importance of the functional group of annual plant species (with early and fast growing characteristics), which contribute to biotic resistance against invasion by *P. australis* (Byun et al., 2013), *Ageratina altissima* (Byun & Lee, 2017), and *Sicyos angulatus* (Byun, Oh, et al., 2020); see also the case of *Taeniatherum caput‐medusae* (Sheley & James, 2017). A recent study also showed that early germination of native seeds and early creation of canopy cover are important characteristics for improving biotic resistance to invasive species (Byun, 2022), consistent with the findings of this study (Figure 3). Figure 3 shows the role of early canopy cover formation and rapid shoot growth, presented (indicated by plant height) in suppressing the growth of invasive species.

This study hypothesized that plant species with certain functional traits resist invasion better than other species lacking those traits. However, the effect of species identity on biotic resistance to invasion was not significantly different (Figure 2), except among a few species. The RCI_avg_ of *L. indica* (annual plant; mean = 0.898) was significantly higher than that of *S. sorbifolia* (perennial woody plant; mean = 0.497) in the T3 treatment. The insignificant difference in species variation is consistent with the result of a previous study on *A. trifida* (Byun & Lee, 2018); however, different results were obtained in studies on other invaders with limiting similarity (Byun, Oh, et al., 2020; Hess et al., 2020; Walder et al., 2019). The effectiveness of invasion control was barely 10%–30% (very weak biotic resistance) in previous studies and 70%–100% in the current study when *A. trifida* arrived 6 weeks later after native species. A substantial control of plant invasion is possible if the seeds of native species are sown well before the arrival of an invasive plant species in a natural habitat that has never been invaded. The concept of sowing native seeds 6 weeks earlier in the growing season is also supported by the results of previous studies, which employed the priority effects of native species or restoring of native plant communities after invasion control (Young et al., 2017). This strategy can provide ample space and time to natives for creating a strong competition for the late‐arriving invasive species, consistent with the results of the current study. The concomitant arrival of invasive species and native species may suppress the growth of native communities. In such cases, *A. trifida* outcompetes most native species (as shown in the T1 treatment in this study).

Aboveground biomass and canopy cover were identified as the most important plant performance traits in this study (Figure 4). The biomass of resident communities has been documented as one of the best indicators of competitive ability of native species (Gaudet & Keddy, 1988) and their level of biotic resistance to invasive species (Byun et al., 2013; Byun, Choi, & Kang, 2020; Byun & Lee, 2017; Byun, Oh, et al., 2020; Lulow, 2006). Early sowing of native seeds significantly increased native canopy cover and biomass in this study, which is inconsistent with the results of a previous study (Tarsa et al., 2022). A canopy complexity, comprising the canopies of different species, can enhance canopy cover and suppress invaders (Lindig‐Cisneros & Zedler, 2002). High biomass of resident species implies reduced resource availability to invaders, leading to strong biotic resistance, according to the fluctuating resource availability hypothesis (Davis et al., 2000).

Responses of invasive and native species to various environmental conditions and interactions are important in determining the success of invasion (Berg et al., 2016; Byun et al., 2015, 2018, 2022; Parepa et al., 2013; Rohal et al., 2019). In this study, environmental conditions optimal for invasion by *A. trifida* (fertile bare ground) were created. A recent study examined the environmental influence of flooding regimes and fertility on *A. trifida*, and concluded that maintaining an appropriate water regime and avoiding eutrophication in wetlands would be necessary to prevent *A. trifida* invasion (Park et al., 2019). In grasslands with relatively high water availability and strong interspecific competition, *A. trifida* is much more abundant than *A. artemisiifolia* over the years (Dong et al., 2020). In the case of invasion by *S. angulatus*, the interaction between native species and soil fertility is also an important determinant of biotic resistance to invasion (Byun et al., 2022).

### 4.1 Implications for application

The results of this study suggest that restoration practices such as sowing native seeds early can reduce invasive species like *A. trifida*. Additionally, the season of sowing also plays a critical role in the success of *A. trifida*. Native seeds sown very early in the spring (e.g., in mid‐February, which coincides with the spontaneous timing of emergence of *A. trifida* in its natural habitat in South Korea) effectively suppressed invasion by *A. trifida*. It is also important to select native species with early germination. Annual plant species usually tend to germinate and establish earlier than long‐living perennials (personal observation). However, in order to avoid the gradual loss of annuals, repeated sowing of the seeds of annuals might be needed.

Given that the arrival of native seeds 6 weeks earlier than those of invasive species in this study is an artificial scenario, the selective eradication of *A. trifida* plants (e.g., by leaving behind alive native plants) can be an effective measure of controlling *A. trifida*. The germination of invasive seeds present in the seed bank may be suppressed by the available native vegetation. Eradicating *A. trifida* at the vegetative stage and removing newly emerging propagules during the germination season will additionally support native plant communities. Mowing all species (both invasive alien species and other native species) will not be effective, since there will be no biotic resistance left to control reinvasion by invasive alien species such as *A. trifida* (Nagy et al., 2022). The eradication control method might need to be repeated to deplete the soil bank of invasive seeds and eliminate the legacy impact on soil seed bank dynamics (Wang et al., 2022). Further research is required to confirm the implications of this study for restoration.

## AUTHOR CONTRIBUTIONS

**Chaeho Byun:** Conceptualization (equal); data curation (equal); formal analysis (equal); funding acquisition (equal); investigation (equal); methodology (equal); project administration (equal); resources (equal); software (equal); supervision (equal); validation (equal); visualization (equal); writing – original draft (equal); writing – review and editing (equal).

## CONFLICT OF INTEREST STATEMENT

The author declares no conflict of interest.

### OPEN RESEARCH BADGES

This article has earned an Open Data badge for making publicly available the digitally‐shareable data necessary to reproduce the reported results. The data is available at [https://doi.org/10.6084/m9.figshare.22236655.v1].

## Supporting information


Appendix S1.

## Data Availability

The data supporting the results were archived in the following public repository (Figshare). https://doi.org/10.6084/m9.figshare.22236655.v1.
