# Supplementary figures and images for: Role of priority effects in invasive plant species management: Early arrival of native seeds guarantees the containment of invasion by Giant ragweed
Source: Ecol Evol. 2023 Mar 26;13(3):e9940. doi: 10.1002/ece3.9940 (PMC10040727; doi:10.1002/ece3.9940)

Supporting information

Appendix S1.


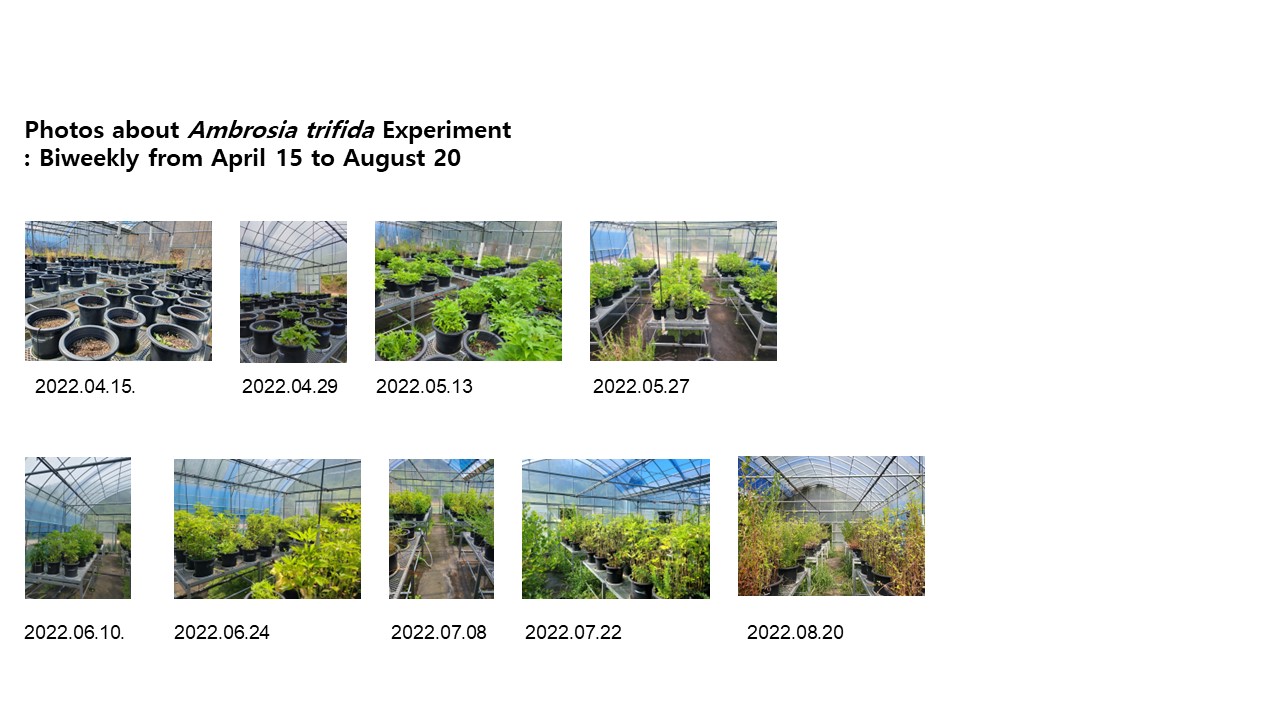

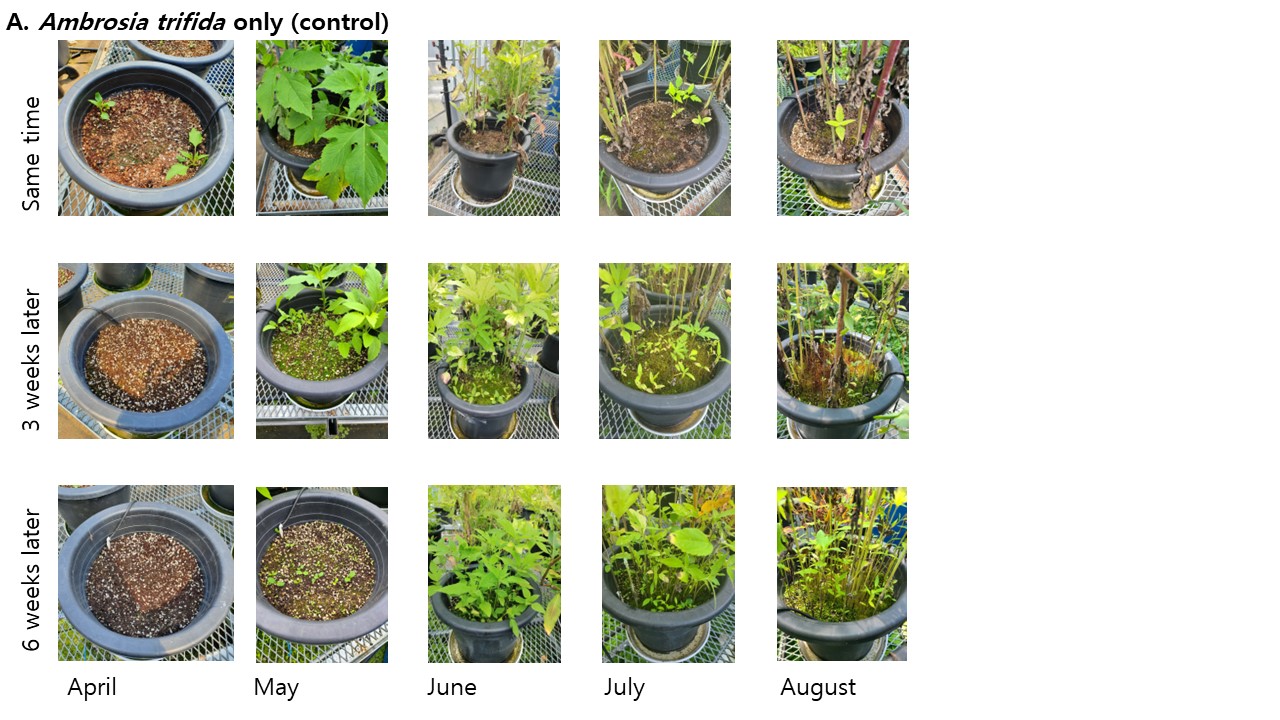


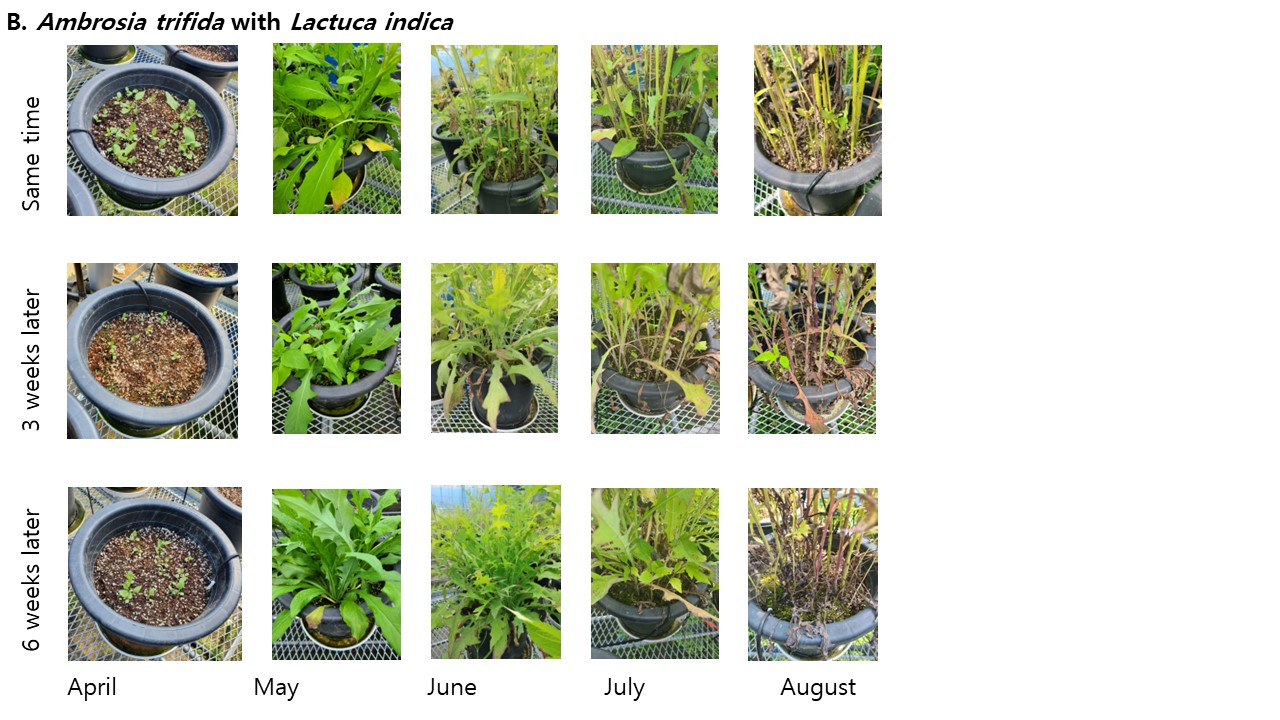


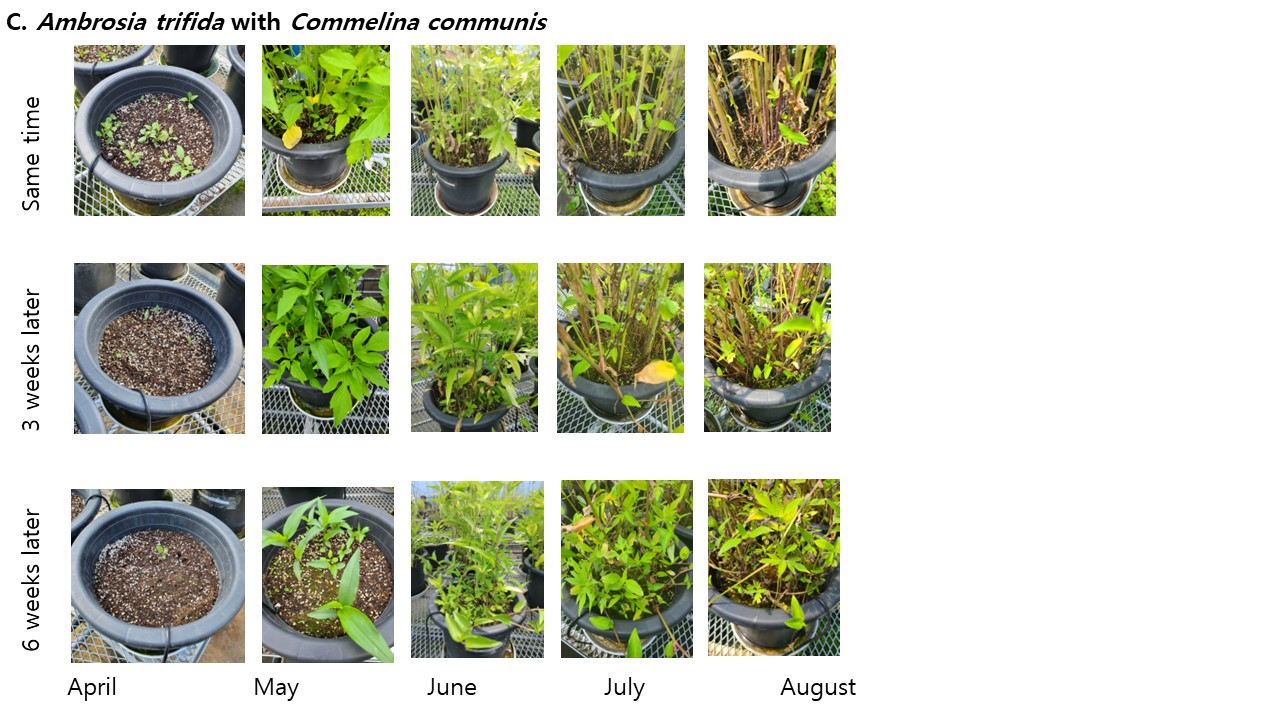


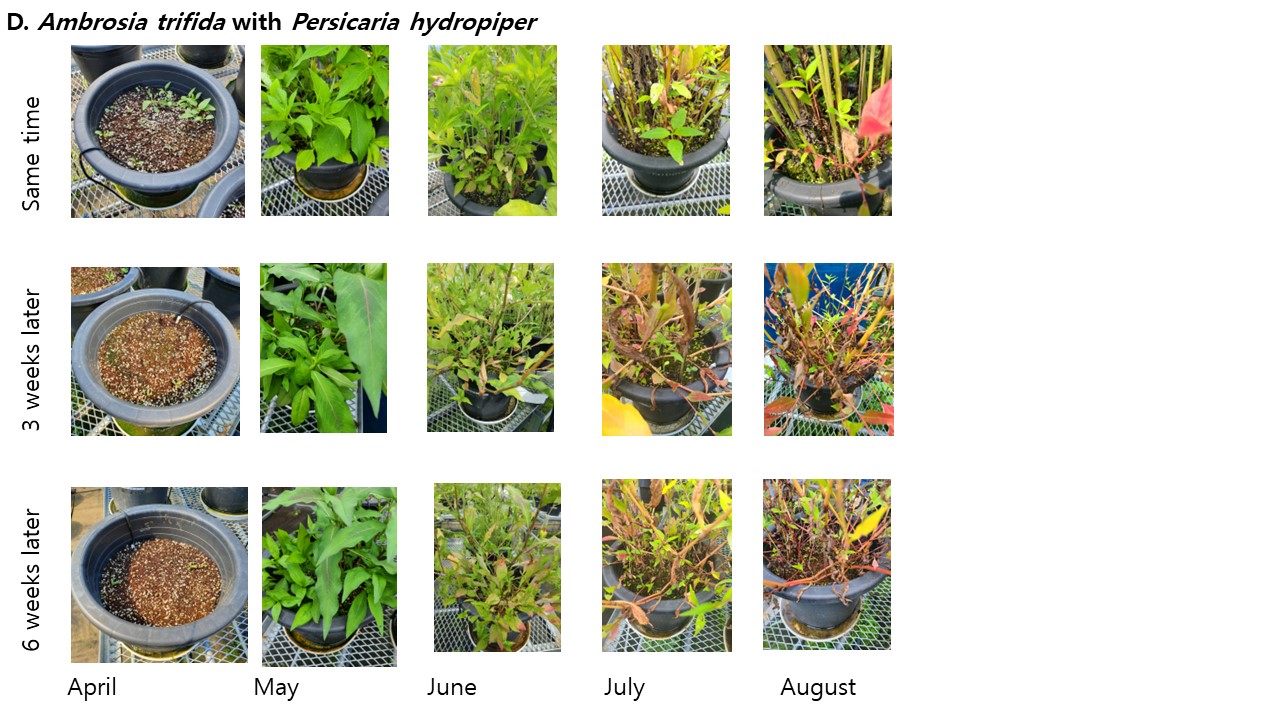


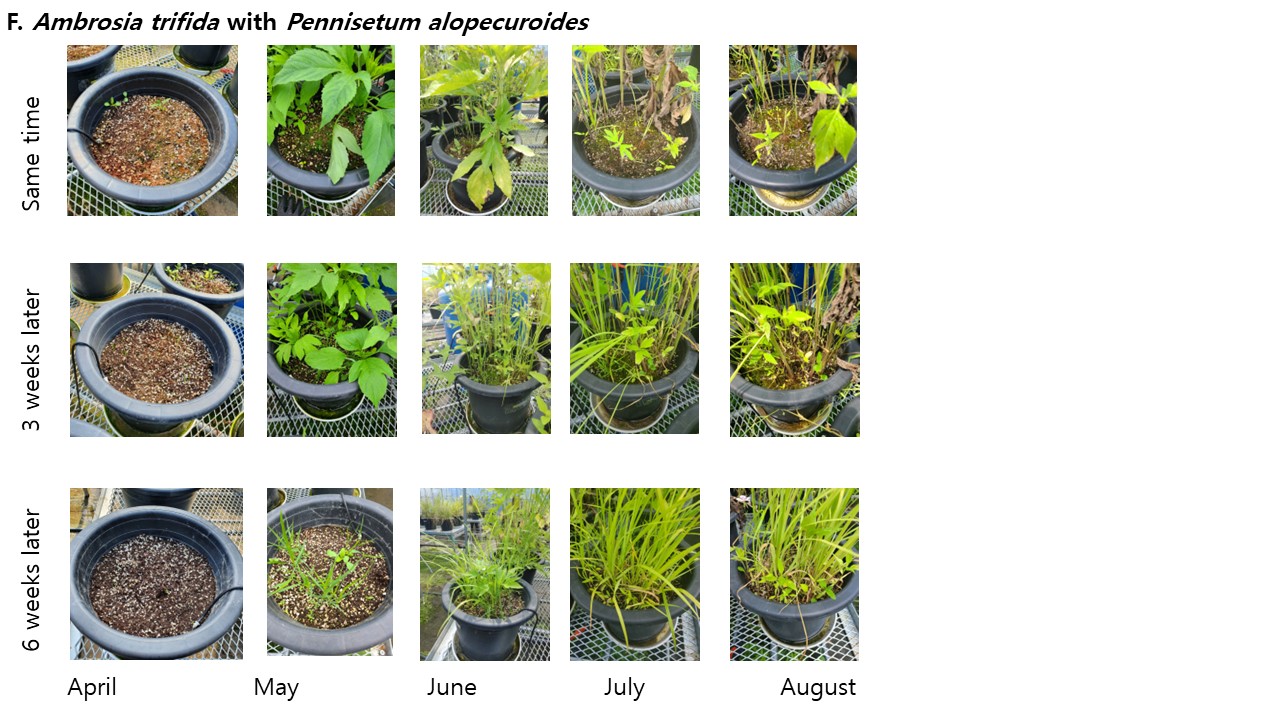


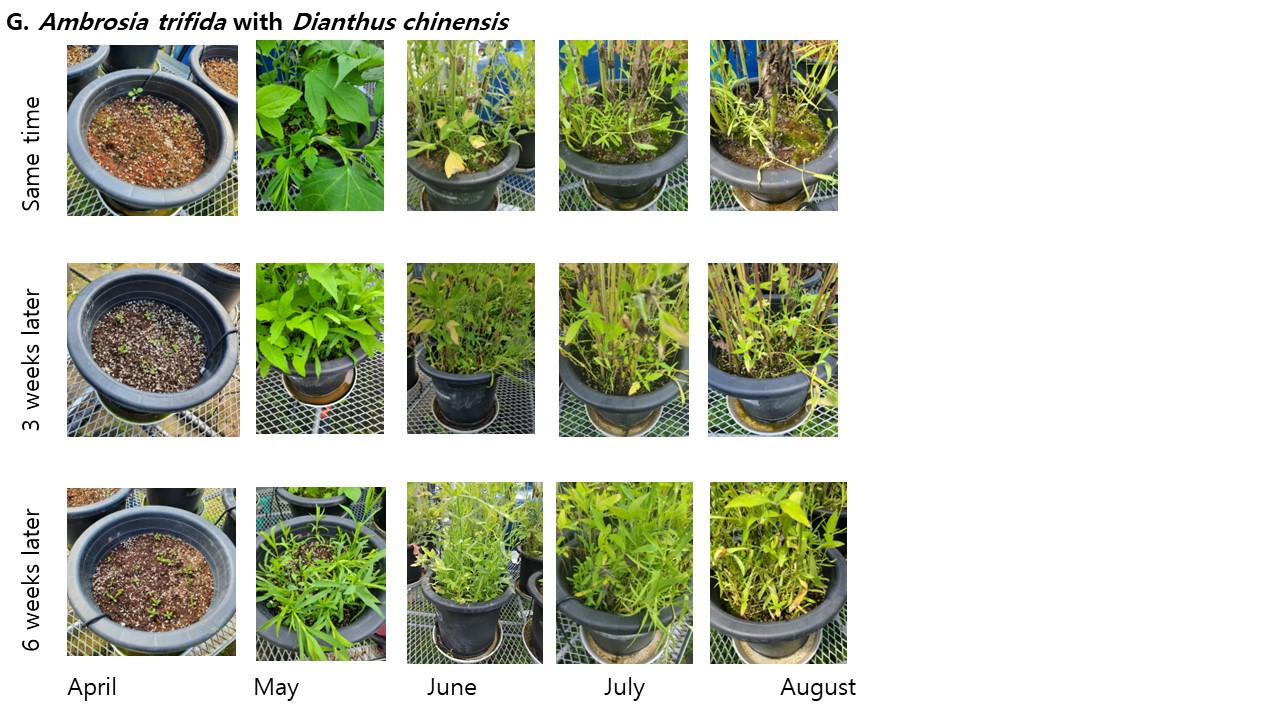


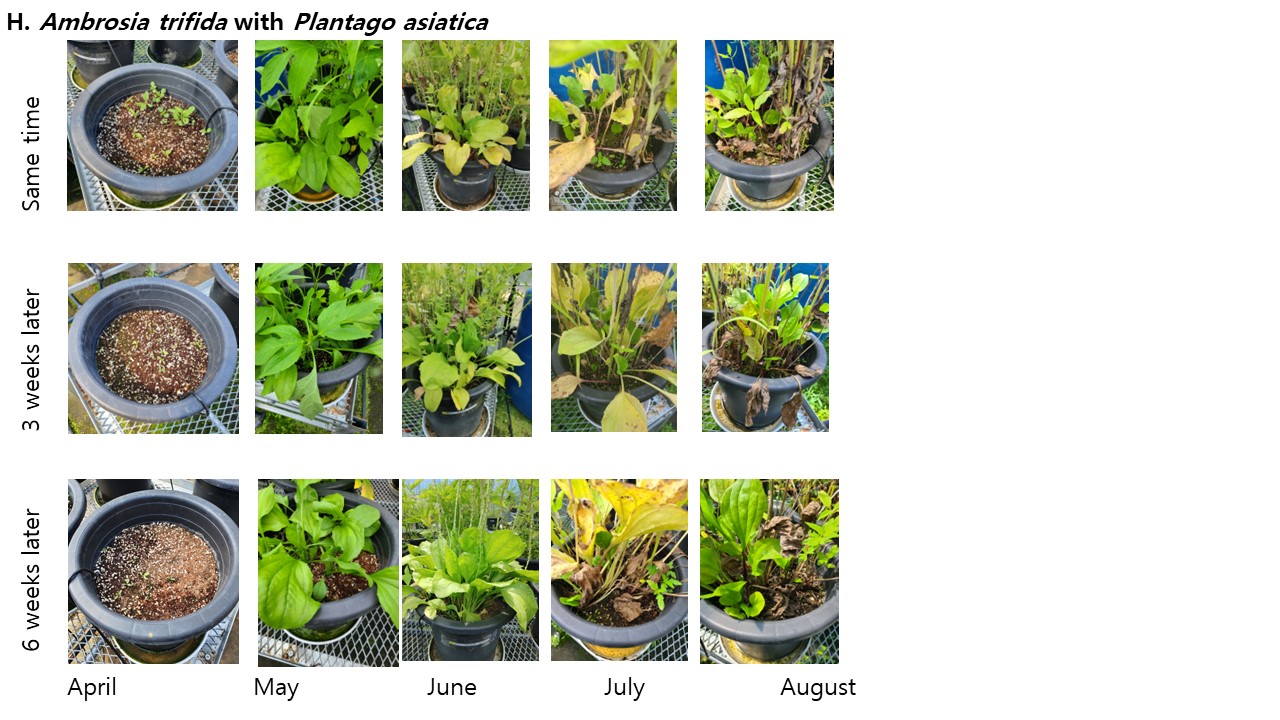


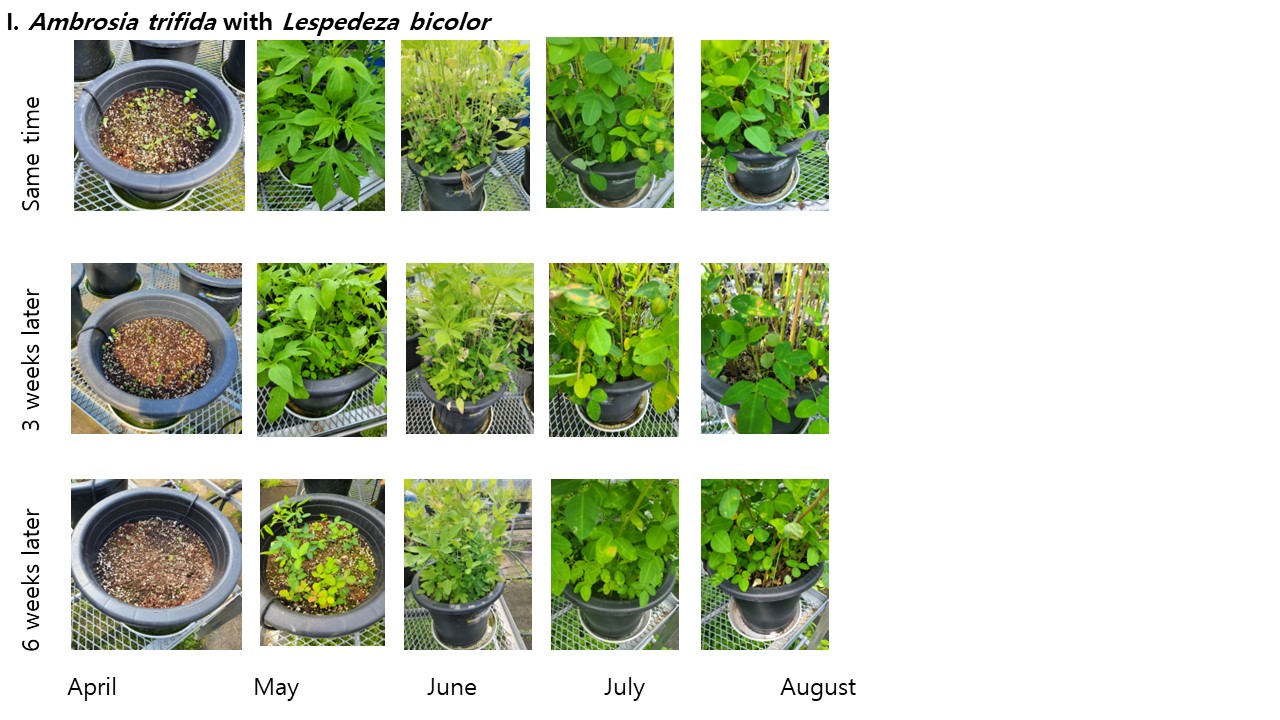

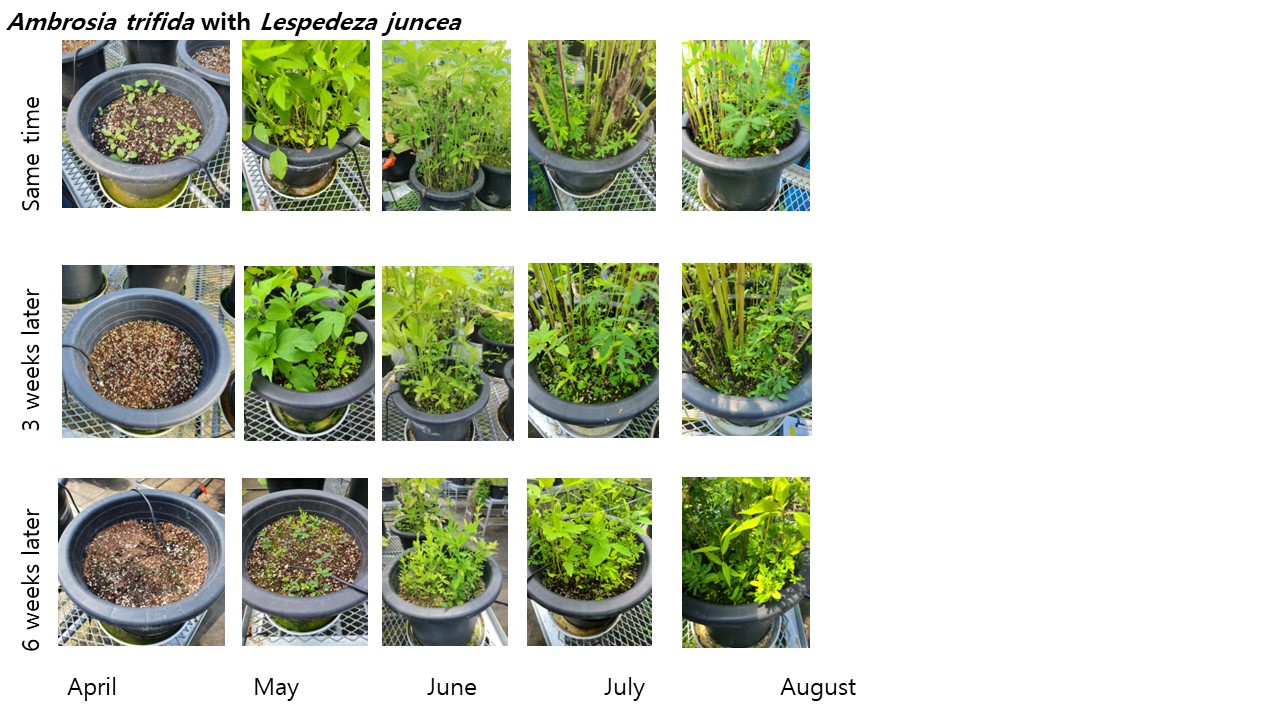


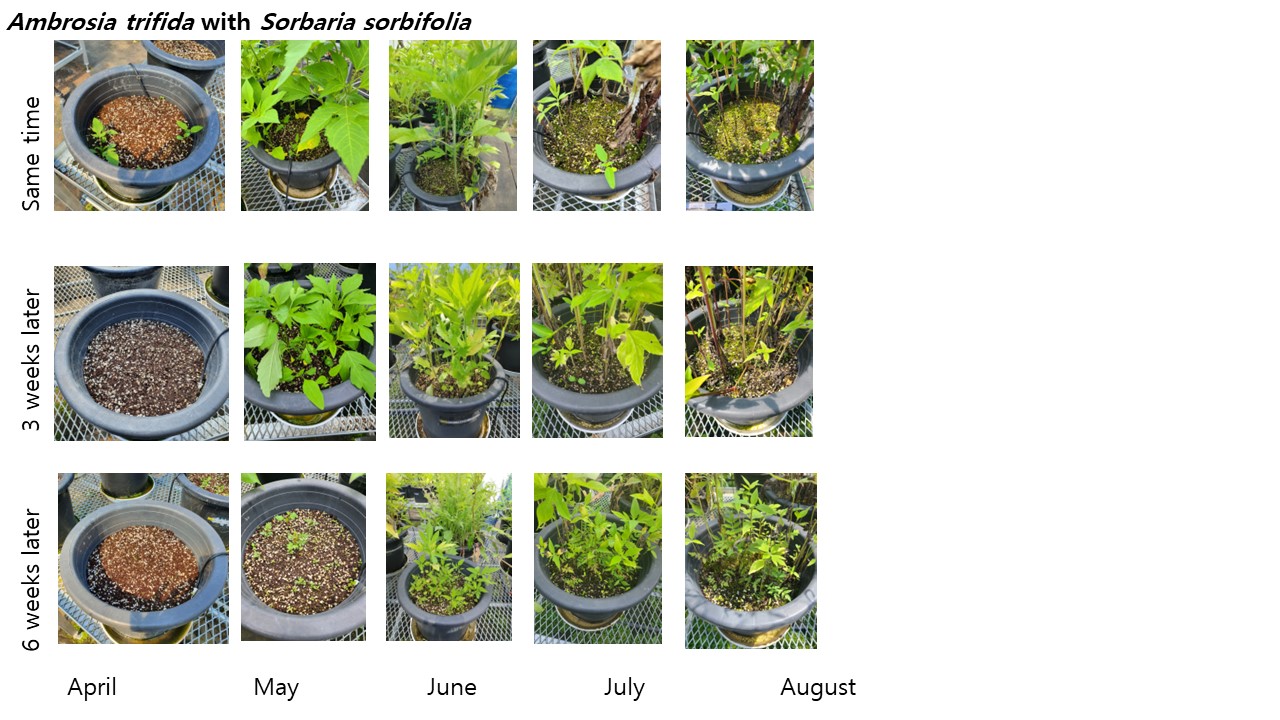

Supplement: Supplementary file 1 — Appendix S1. [file ECE3-13-e9940-s001.docx]
